# Cell type-specific activation of mitogen-activated protein kinase in D1 receptor-expressing neurons of the nucleus accumbens potentiates stimulus-reward learning in mice

**DOI:** 10.1038/s41598-018-32840-1

**Published:** 2018-09-26

**Authors:** Md. Ali Bin Saifullah, Taku Nagai, Keisuke Kuroda, Bolati Wulaer, Toshitaka Nabeshima, Kozo Kaibuchi, Kiyofumi Yamada

**Affiliations:** 1 Department of Neuropsychopharmacology and Hospital Pharmacy, Nagoya University Graduate School of Medicine, Nagoya, 466-8560 Japan; 2 Department of Cell Pharmacology, Nagoya University Graduate School of Medicine, Nagoya, 466-8560 Japan; 3 Advanced Diagnostic System Research Laboratory Fujita Health University, Graduate School of Health Sciences, Toyoake, 470-1192 Japan

## Abstract

Medium spiny neurons (MSN) in the nucleus accumbens (NAc) are a fundamental component of various aspects of motivated behavior. Although mitogen-activated protein kinase (MAPK) signaling plays a crucial role in several types of learning, the cell type-specific role of MAPK pathway in stimulus-reward learning and motivation remains unclear. We herein investigated the role of MAPK in accumbal MSNs in reward-associated learning and memory. During the acquisition of Pavlovian conditioning, the number of phosphorylated MAPK1/3-positive cells was increased significantly and exclusively in the NAc core by 7-days of extensive training. MAPK signaling in the respective D1R- and D2R-MSNs was manipulated by transfecting an adeno-associated virus (AAV) plasmid into the NAc of *Drd1a-Cre* and *Drd2-Cre* transgenic mice. Potentiation of MAPK signaling shifted the learning curve of Pavlovian conditioning to the left only in *Drd1a-Cre* mice, whereas such manipulation in D2R-MSNs had negligible effects. In contrast, MAPK manipulation in D2R-MSNs of the NAc core significantly increased motivation for food rewards as found in *Drd1a-Cre* mice. These results suggest that MAPK signaling in the D1R-MSNs of NAc core plays an important role in stimulus-reward learning, while MAPK signaling in both D1R- and D2R-MSNs is involved in motivation for natural rewards.

## Introduction

Actions and responses to reward-associated stimuli are essential components in daily life. Individual behavior to reward-associated stimuli may be analyzed using Pavlovian conditioning and instrumental conditioning^1,2^. In Pavlovian conditioning, a neutral stimulus becomes a conditioned stimulus (CS) through a concomitant presentation with an unconditioned stimulus (US). Associative learning between US and CS promotes approaches toward the site of US and/or CS during CS presentation. The approach and interaction with the CS are called sign-tracking^3^, whereas the approach to the location of US delivery is defined as goal-tracking^4^. In instrumental conditioning, an animal is trained to make actions by delivering rewards^2^. Learning of the action-outcome association is acquired through the consequence of the action. It is well known that dopamine (DA) signaling plays a crucial role in both types of learning^5–7^ and dysfunctions in dopaminergic neurons contribute to the motivational symptoms observed in the pathologies of psychiatric disorders including depression, schizophrenia, parkinsonism and other disorders^8–10^.

Among the brain regions involved in the reward circuit, the nucleus accumbens (NAc) is of particular interest in reward-associated learning because of its involvement in the affective and motivational aspects of behavior^11,12^. Most neurons in the NAc are inhibitory GABAergic medium spiny neurons (MSNs), which have been separated into two populations, DA D1 receptor (D1R)- and DA D2 receptor (D2R)-expressing MSNs. Genetic and pharmacological approaches have been used to clarify the respective roles of D1R and D2R signaling in different aspects of reward-related behavior^13–18^. For example, the inactivation of D1R- and D2R-MSNs impaired reward-associated learning and aversive learning, respectively^14^. A microinjection of a D1R antagonist into the NAc reduced Pavlovian instrumental transfer. D2R antagonism also showed similar, but less pronounced effects^15^.

One of the common signaling pathways that are modulated by D1Rs and D2Rs is mitogen-activated protein kinase (MAPK, also known as ERK) signaling. Our recent study demonstrated that the stimulation of D1Rs phosphorylates MAPK1/3 (also known as ERK1/2) through the PKA/Rasgrp2/Rap1 pathway^19^. The activation of MAPK1/3 enhances the spike firing of D1R-MSNs in response to excitatory glutamatergic inputs, eventually potentiating reward-associated behavioral outputs^20^. In contrast, the stimulation of D2Rs is known to inhibit the activity of the MAPK signaling pathway^21,22^. In animal models, exposure to a cue previously associated with a reward outcome, such as food and drug, resulted in the activation of MAPK signaling in the NAc and the blockade of this signaling pathway impaired reward-associated memory^23,24^. These findings indicate the necessity of accumbal MAPK signaling in stimulus-reward learning. Although the role of dopaminergic receptors and MAPK signaling in food reward-associated instrumental tasks have been studied^2,24,25^, the precise effects of the cell type-specific activation of MAPK signaling in the NAc on Pavlovian and instrumental conditioning have not yet been clarified.

In the present study, using an adeno-associated virus (AAV)-mediated gene expression technique in targeted cells, we investigated the cell type-specific role of MAPK signaling in accumbal D1R-MSNs and D2R-MSNs in natural reward-associated learning and memory as well as the motivation for rewards. We also investigated their roles in methamphetamine (METH)-associated conditioned place preference (CPP). We herein show that activation of the MAPK pathway in D1R-MSNs, but not D2R-MSNs is important for natural and drug reward-associated learning. Furthermore, our results suggest that the activation of MAPK signaling in D1R-MSNs or D2R-MSNs results in an increase in motivation for rewards.

## Results

### Pavlovian conditioning increases MAPK1/3 phosphorylation exclusively in the NAc core

**Figure 1:**
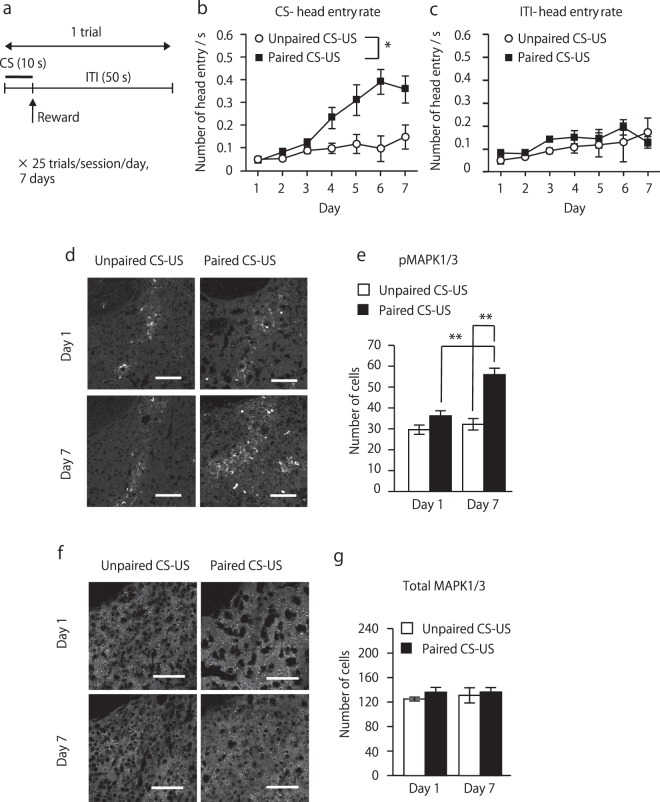
Phosphorylation of MAPK1/3 in the NAc during acquisition of the Pavlovian conditional approach. (**a**) Schematic representation of the Pavlovian conditioning procedure. (**b**) CS-head entry rate in paired CS-US and unpaired CS-US control mice. In unpaired CS-US control group, the timing of food pellet delivery changed randomly within a 60-s trial throughout the session, so that there was no contingency between CS and food reward delivery. (Unpaired CS-US: n = 7; paired CS-US: n = 12). (**c**) Head entry rate during ITI period in C57BL/6 mice (unpaired CS-US: n = 7; paired CS-US: n = 12). (**d**) Confocal images of pMAPK1/3-positive cells in the NAc core after training session in the unpaired CS-US and paired CS-US group. (**e**) Number of pMAPK1/3 in unpaired CS-US and paired CS-US group after training session on day 1 and day 7. (**f**) Confocal images of total MAPK1/3-positive cells in the NAc core. (**g**) Number of total MAPK1/3-positive cells in the NAc core of C57BL/6 mice after the training session of Pavlovian conditioning on days 1 and 7 (Day 1: unpaired CS-US, n = 4; paired CS-US, n = 4; Day 7: unpaired CS-US, n = 4; paired CS-US, n = 5). Data are presented as the mean ± SEM. *p < 0.05. Scale bars represent 100 μm.

In order to analyze the involvement of MAPK signaling within the NAc in natural reward-associated learning, we trained wild-type C57BL/6 mice in food reward-associated Pavlovian conditioning (Fig. 1a). In this task, mice learned the association between a reward-predictive cue (light) and food pellet delivery. During the first session on day 1, in the paired CS-US group, head entry rate during CS presentation was minimum. However, it was increased significantly after 7-days of training. On the other hand, the unpaired CS-US control group showed a similar level of head entry after 7-days of training as observed on day 1 (repeated two-way ANOVA, group, F(1, 17) = 8.14, p < 0.05; training, F(6, 102) = 15.52, p < 0.01; group × training interaction, F(6, 102) = 4.68, p < 0.01, Fig. 1b). Head entry rate during inter-trial interval (ITI) period was not significantly different between groups (repeated two-way ANOVA, group, F(1, 17) = 0.89, p = 0.36; training, F(6, 102) = 4.56, p < 0.01, group × training interaction, F(6, 102) = 0.63, p = 0.71, Fig. 1c). To examine changes in an endogenous phosphorylation level of MAPK1/3, we measured the phosphorylated MAPK1/3-positive cells immediately after the behavioral test. On day 1, there was no significant difference in the number of pMAPK1/3-positive cells in the NAc core between the paired and unpaired CS-US groups. On day 7, the number of pMAPK1/3-positive cells in the paired CS-US group was markedly increased as compared to the respective cell number in the same group on day 1 and in the unpaired CS-US group on day 7 (two-way ANOVA, day, F(1, 23) = 24.29, p < 0.01, training, F(1, 23) = 20.42, p < 0.01, day × training, F(1, 23) = 6.15, p < 0.05; Figs 1d,e and S1). This increase in pMAPK1/3-positive cells was only observed in the core, not in the shell of the NAc (two-way ANOVA, day, F(1, 16) = 8.51, p < 0.05, region, F(1, 16) = 82.06, p < 0.01, gene × region, F(1, 16) = 3.58, p = 0.08, Fig. S2a,b). A significant correlation was evident between the number of pMAPK1/3-positive cells in the NAc core and CS head entry (Pearson correlation analysis, p < 0.01, Fig. S2c). The number of pMAPK1/3-positive cells in the unpaired CS-US control group did not increase after 7-days training (Fig. 1d,e). The number of total MAPK1/3-positive cells was not different between two groups on either day 1 or day 7 (two-way ANOVA, day, F(1, 13) = 0.17, p = 0.69; training, F(1, 13) = 0.96, p = 0.35; day × training, F(1, 13) = 0.11, p = 0.75, Figs 1f,g and S3).

### Relationship between D1R-MSNs and D2R-MSNs in the NAc core with natural reward-related learning

**Figure 2:**
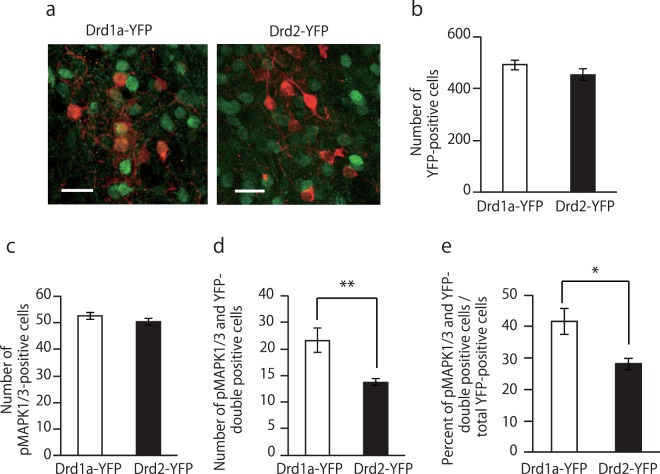
Proportion of phosphorylated MAPK1/3-positive cells in the NAc core of Pavlovian-conditioned mice. (**a**) Representative confocal images of pMAPK1/3-positive cells in the NAc core of *Drd1a-YFP* and *Drd2-YFP* mice after the training session of Pavlovian conditioning on day 7. (**b**–**d**) Quantification of YFP-positive (**b**), pMAPK1/3-positive (**c**) and double-positive (**d**) cells in each mouse line. (**e**) Percentage of pMAPK1/3 and YFP double-positive cells in all pMAPK1/3-positive cells in each mouse line (n = 4 for *Drd1a-YFP* and n = 5 for *Drd2-YFP*). Data are presented as the mean ± SEM. *p < 0.01, **p < 0.01. Scale bars represent 25 μm.

To further clarify which subtype of MSN is involved in MAPK activation in the NAc core after the training session, both *Drd1a-YFP* and *Drd2-YFP* mice were used for Pavlovian conditioning. We collected brain samples of these transgenic mice after the 7^th^ day training session because the paired CS-US group showed the highest level of MAPK1/3 phosphorylation at this time point. No significant differences were observed in the total number of YFP- and pMAPK1/3-positive cells in the NAc core between *Drd1a-YFP* and *Drd2-YFP* mice (non-paired Student’s *t*-test; YFP-positive cells, t(7) = 2.36, p = 0.23; pMAPK1/3-positive cells, t(7) = 2.36, p = 0.23, Figs 2a–c and S4). However, the number of cells co-expressing pMAPK1/3 and YFP in the NAc core was significantly higher in *Drd1a-YFP* mice than in *Drd2-YFP* mice (non-paired Student’s *t*-test, t(7) = 2.36, p < 0.01, Fig. 2d). After the 7^th^ training session, 41% of pMAPK1/3-positive cells were D1R-MSNs, while only 28% of cells were D2R-MSNs (non-paired Student’s *t*-test, t(7) = 2.36, p < 0.05, Fig. 2e).

### Manipulation of MAPK signaling in accumbal D1 and D2-MSNs

**Figure 3:**
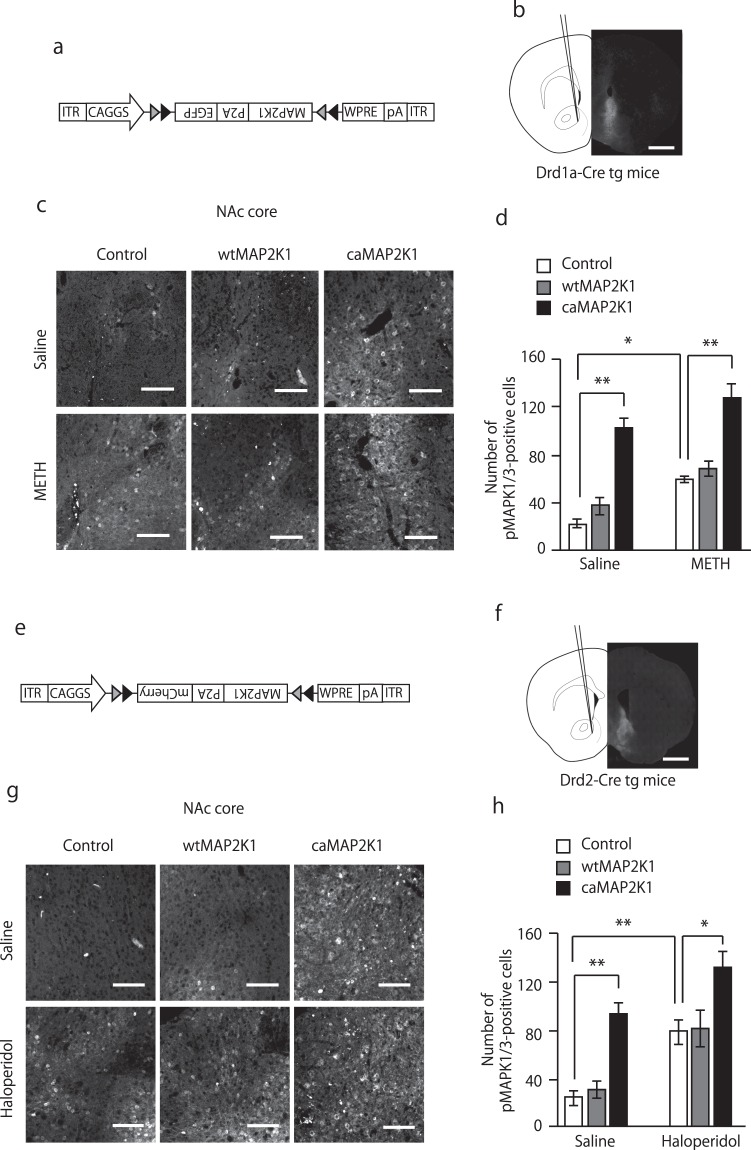
Manipulation of MAPK signaling in accumbal D1R and D2R-MSNs. (**a**) Schematic diagram shows the AAV construct. (**b**) Stereotaxic injection of AAVs into the NAc of *Drd1a-Cre* transgenic mice and representative coronal brain slices showing the expression of EGFP (shown in grey) 3 weeks after the AAV injection. The scale bar represents 1 mm. (**c**,**d**) pMAPK1/3-positive cells in the NAc of AAV-mutant MAP2K1-injected *Drd1a-Cre* transgenic mice. *Drd1a-Cre* transgenic mice were microinjected with AAV-mutant MAP2K1 into the NAc. Three weeks after the treatment, mice were administered saline or METH (10 mg/kg, i.p.) 30 minutes before perfusion. (**c**) Representative confocal images of pMAPK1/3. The upper panel represents saline-treated mice. The lower panel represents METH-treated mice. Scale bars represent 100 μm. (**d**) Quantification of pMAPK1/3-positive cells in the NAc core (n = 5 for the saline-treated control, n = 3 for saline-treated wtMAP2K1, n = 3 for saline-treated caMAP2K1, n = 4 for the METH-treated control, n = 3 for METH-treated wtMAP2K1, n = 4 for METH-treated caMAP2K1). (**e**) Schematic diagram shows the AAV construct. (**f**) Stereotaxic injection of AAVs into the NAc of *Drd2-Cre* transgenic mice and representative coronal brain slices showing the expression of mCherry (shown in grey) 3 weeks after the AAV injection. The scale bar represents 1 mm. (**g**,**h**) pMAPK1/3-positive cells in the NAc of AAV-mutant MAP2K1-injected *Drd2-Cre* transgenic mice. *Drd2-Cre* transgenic mice were microinjected with AAV-mutant MAP2K1 into the NAc. Three weeks after the treatment, mice were administered saline or haloperidol (0.5 mg/kg, i.p.) 15 minutes before perfusion. (**g**) Representative confocal images of pMAPK1/3. The upper panel represents saline-treated mice. The lower panel represents haloperidol-treated mice. Scale bars represent 100 μm. (**h**) Quantification of pMAPK1/3-positive cells in the NAc core (n = 5 for the saline-treated control, n = 3 for saline-treated wtMAP2K1, n = 3 for saline-treated caMAP2K1, n = 4 for the haloperidol-treated control, n = 3 for haloperidol-treated wtMAP2K1, n = 3 for haloperidol-treated caMAP2K1). Data are presented as the mean ± SEM. *p < 0.01, **p < 0.01.

In order to manipulate MAPK signaling in D1R-MSNs, we first injected AAV-Flex-EGFP-MAP2K1 [wild-type MAP2K1 (wtMAP2K1) or constitutively active mutant MAP2K1 (caMAP2K1, the phospho-mimic S218D and S222E MAP2K1)]^26^ into the NAc of *Drd1a-Cre* transgenic mice (Fig. 3a,b). We assessed the activity of the MAP2K1 gene by examining the number of phosphorylated MAPK1/3-positive cells. MAPK1/3 is the downstream molecular target of MAP2K1. Mice were divided into two groups. One group of *Drd1a-Cre* mice received saline, while the other received METH (10 mg/kg, i.p.) 30 min before perfusion in order to stimulate endogenous DA release. Luo *et al*.^27^ have demonstrated that acute cocaine induces fast activation of D1R-MSNs and progressive deactivation of D2R-MSNs. Similarly, acute METH injection should also induce phasic dopamine release and subsequently activate D1R-MSNs rapidly. In the NAc core, caMAP2K1 expression resulted in more pMAPK1/3-positive cells than in the control group of saline-treated *Drd1a-Cre* mice (Figs 3c,d and S5). Acute METH treatment significantly increased the number of pMAPK1/3-positive cells in control mice, which was further potentiated by caMAP2K1 expression in METH-treated *Drd1a-Cre* mice (two-way ANOVA, gene, F(2, 16) = 62.10, p < 0.01, treatment, F(1, 16) = 26.56, p < 0.01, gene × treatment interaction, F(2, 16) = 0.34, p = 0.71, Figs 3c,d and S5). A similar pattern of changes in the number of pMAPK1/3-positive cells was observed in the NAc shell in *Drd1a-Cre* mice (two-way ANOVA, gene, F(2, 16) = 22.35, p < 0.01, treatment, F(1, 16) = 8.69, p < 0.01, gene × treatment interaction, F(2, 16) = 1.19, p = 0.33, Fig. S6a).

In order to manipulate MAPK signaling in D2R-MSNs, *Drd2-Cre* mice were injected with AAV vectors containing mutant MAP2K1 (Fig. 3e,f). *Drd2-Cre* mice were injected with the D2R antagonist haloperidol (0.5 mg/kg, i.p.), which has been shown to stimulate MAPK1/3 phosphorylation in MSNs 15 min after its administration^28^. caMAP2K1 transfection significantly increased the number of pMAPK1/3-positive cells in the NAc core of saline-treated *Drd2-Cre* mice (two-way ANOVA, gene, F(2, 15) = 26.00, p < 0.01, treatment, F(1, 15) = 39.83, p < 0.01, gene × treatment interaction, F(2, 15) = 0.45, p = 0.65, Figs 3g,h and S7). Haloperidol-induced increases in the number of pMAPK1/3-positive cells were potentiated in the NAc core of caMAP2K1-transfected *Drd2-Cre* mice (Figs 3g,h and S7). In the NAc shell, expression of caMAP2K1 significantly increased the number of pMAPK1/3-positive cells in the saline-treated *Drd2-Cre* mice (Fig. S6b). caMAP2K1 expression failed to potentiate haloperidol-induced MAPK1/3 hyperphosphorylation, probably due to a ceiling effect by the drug treatment (two-way ANOVA, gene, F(2, 15) = 21.30, p < 0.01, treatment, F(1, 15) = 46.75, p < 0.01, gene × treatment interaction, F(2, 15) = 4.83, p < 0.05, Fig. S6b).

### Hyperactivity of MAPK signaling in accumbal D1R-MSNs but not in D2R-MSNs potentiates learning and conditioned approaches in Pavlovian conditioning

**Figure 4:**
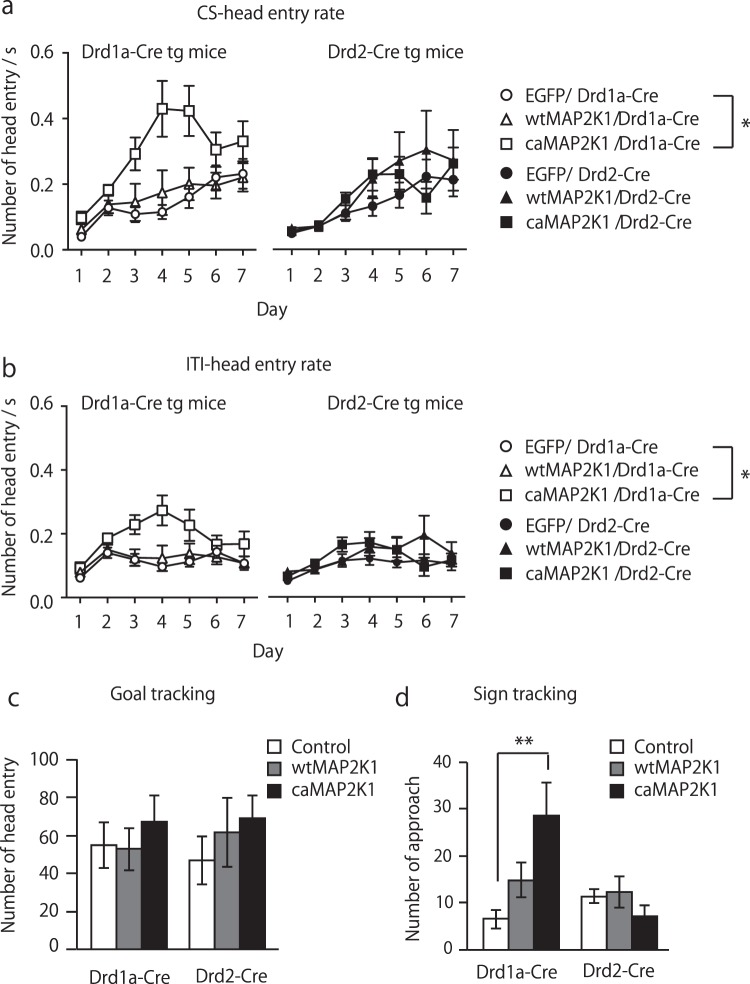
Hyperactivity of MAPK signaling in accumbal D1R-MSNs, but not in D2R-MSNs potentiates learning in Pavlovian conditioning. (**a**,**b**) Head entry rate during CS presentation (**a**) and ITI (**b**) period in mutant MAP2K1-transfected *Drd1a-Cre* and *Drd2-Cre* mice. (**c**,**d**) Number of conditional approaches for goal-tracking (**c**) and sign-tracking (**d**) behaviors in mutant MAP2K1-transfected *Drd1a-Cre* and *Drd2-Cre* mice on day 7 (n = 9 for the control/*Drd1a-Cre*, n = 13 for wtMAP2K1/*Drd1a-Cre*, n = 8 for caMAP2K1/*Drd1a-Cre*, n = 11 for the control/*Drd2-Cre*, n = 9 for wtMAP2K1/*Drd2-Cre*, n = 12 for caMAP2K1/*Drd2-Cre*). Data are presented as the mean ± SEM. *p < 0.01, **p < 0.01.

We then investigated whether genetic manipulations of MAPK activity in accumbal D1R-MSNs affected natural reward-associated learning in Pavlovian conditioning. The learning curve in caMAP2K1-transfected *Drd1a-Cre* mice significantly shifted to the left from those in control and wtMAP2K1-transfected *Drd1a-Cre* mice and from all virus injected groups of *Drd2-Cre* mice (repeated two-way ANOVA; gene, F(5, 56) = 2.97, p < 0.05; day, F(6, 328) = 24.70, p < 0.01; gene × day, F(30, 328) = 1.44, p = 0.07; Fig. 4a). Head entry rate during ITI period also showed a significant increase in the *Drd1a-Cre* caMAP2K1 group compared to other groups (repeated two-way ANOVA; gene, F(5, 56) = 3.30, p < 0.05; day, F(6, 328) = 10.51, p < 0.01; gene × day, F(30, 328) = 1.54, p < 0.05; Fig. 4b). Head entry rate for caMAP2K1-transfected *Drd1a-Cre* mice was significantly higher during CS presentation compared to ITI period (repeated two-way ANOVA, gene, F(1, 14) = 5.89, p < 0.05, day, F(6, 84) = 9.44, p < 0.01, gene × day, F(6, 84) = 2.08, p = 0.06, Fig. S8).

Previous studies have shown that dopamine differentially modulates the conditioned responses during Pavlovian conditioning^6^ and activation of D1 receptors in the NAc core facilities assignment of incentive salience to reward cue^2^. Therefore, we analyzed the behavioral subtype of conditional responses in the last conditioning session. No changes in goal-tracking or sign-tracking behavior were evident between unpaired and paired CS-US C57BL/6 mice (sign-tracking, non-paired Student’s *t*-test, t(11) = 2.2, p = 0.41; goal-tracking, t(11) = 2.20, p = 0.12, Fig. S9a,b). Under this experimental condition, goal-tracking behavior showed no changes among all the groups of *Drd1a-* and *Drd2-Cre* mice (two-way ANOVA, virus gene, F(2, 52) = 0.94, p = 0.40; mouse genotype, F(1, 52) = 0.01, p = 0.94; virus gene × mouse genotype, F(2, 52) = 0.20, p = 0.82, Fig. 4c), whereas caMAP2K1-transfected *Drd1a-Cre* mice exhibited a significant increase in sign-tracking behaviors (two-way ANOVA; virus gene, F(2, 52) = 2.1, p = 0.13; mouse genotype, F(1, 52) = 4.60, p < 0.05; virus gene × mouse genotype, F(2, 52) = 6.20, p < 0.01, Fig. 4d).

### Hyperactivation of MAPK signaling in accumbal D1R-MSNs and D2R-MSNs increases motivation for rewards in an instrumental task

**Figure 5:**
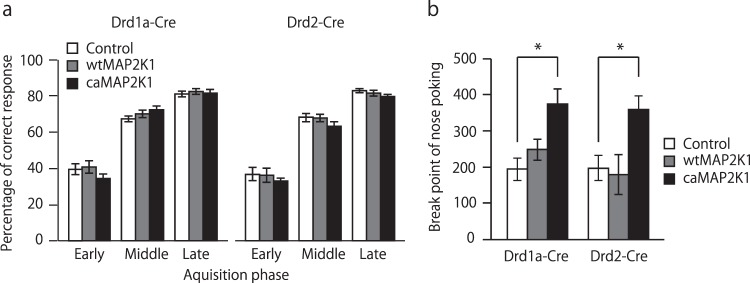
Hyperactivity of MAPK signaling in accumbal D1R-MSNs and D2R-MSNs increases motivation for food rewards but not instrumental learning. (**a**) Percentage of correct responses during the acquisition phase of the FR2 random nose-poking task in AAV-mutant MAP2K1-microinjected *Drd1a-Cre* and *Drd2-Cre* mice. (**b**) Break point of nose poking during the progressive ratio task in AAV-mutant MAP2K1-microinjected *Drd1a-Cre* and *Drd2-Cre* mice (n = 9 for the control/*Drd1a-Cre*, n = 13 for wtMAP2K1//*Drd1a-Cre*, n = 8 for caMAP2K1/*Drd1a-Cre*, n = 11 for the control/*Drd2-Cre*, n = 9 for wtMAP2K1/*Drd2-Cre*, n = 12 for caMAP2K1/*Drd2-Cre*). Data are presented as the mean ± SEM. *p < 0.05.

In instrumental conditioning, mice learn the association between performance of a specific task and its consequence. In the present study, the response (nose poking) requirement for food reward is indicated by a cue that has previously indicated reward delivery in Pavlovian learning. Increased behavioral approaches towards CS in Pavlovian conditioning suggest that caMAP2K1-transfected *Drd1a-Cre* mice have a greater ability to comprehend CS-reward association. Therefore, we next investigated whether caMAP2K1 gene transfection could improve operant learning or not. In the FR2 random nose-poking task, overexpression of mutant MAP2K1 gene in the NAc of *Drd1a-Cre* and *Drd2-Cre* mice failed to influence instrumental learning and performance (two-way ANOVA; gene, F(4, 57) = 1.32, p = 0.27, training, F(2, 114) = 618.05, p < 0.01, gene × training, F(8, 114) = 0.50, p = 0.86, Fig. 5a).

An alternative explanation for the increased conditioned approach during Pavlovian conditioning is that the phosphorylation of MAPK1/3 in accumbal D1R-MSNs may be related to the assignment of some value to the CS. We then investigated whether the cell type-specific activation of MAPK in the NAc affects the motivational value of rewards in the progressive ratio task. caMAP2K1 significantly increased the break point of nose poking in *Drd1a-Cre* mice. Unexpectedly, caMAP2K1 expression in accumbal D2R-MSNs, which had no effect on Pavlovian learning, also increased the break point of nose poking (two-way ANOVA, virus gene, F(2, 56) = 11.57, p < 0.01; mouse genotype, F(1, 56) = 0.17, p = 0.68; virus gene × mouse genotype, F(2, 56) = 0.48, p = 0.62, Fig. 5b).

### Effects of MAPK signaling in D1R-MSNs and D2R-MSNs of the NAc on METH-induced CPP

**Figure 6:**
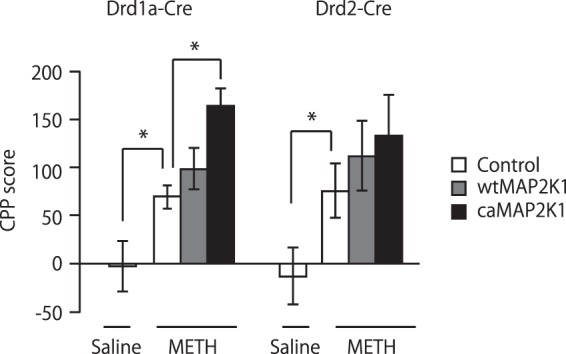
Manipulation of MAPK signaling in accumbal D1R-MSNs controls METH-induced CPP. Effects of the AAV-mediated expression of mutant MAP2K1 on methamphetamine-induced CPP in *Drd1a-Cre* and *Drd2-Cre* mice (n = 16 for the saline-treated control/ *Drd1a-Cre*, n = 18 for the METH-treated control/*Drd1a-Cre*, n = 19 for METH-treated wtMAP2K1/*Drd1a-Cre*, n = 17 for METH-treated caMAP2K1/*Drd1a-Cre*, n = 13 for the saline-treated control/*Drd2-Cre*, n = 10 for the METH-treated control/*Drd2-Cre*, n = 11 for METH-treated wtMAP2K1/*Drd2-Cre*, n = 11 for METH-treated caMAP2K1/*Drd2-Cre*). Data are presented as the mean ± SEM. *p < 0.05.

We also examined the effects of MAPK signaling on drug reward-associated behavioral changes using METH-induced CPP. This paradigm involves the sensory perception of environmental cues, associations between rewards and cues and the approach-inducing actions of a drug^29^. As shown in Fig. 6, METH increased place preference in *Drd1a-Cre* and *Drd2-Cre* mice compared to saline-treated control group (*Drd1a-Cre*, Student’s *t*-test, t(32) = 2.03, p < 0.05; *Drd2-Cre*, Student’s *t*-test, t(21) = 2.09, p < 0.05), which was further potentiated by the expression of caMAP2K1 in the NAc (two-way ANOVA, virus gene, F(2, 80) = 5.26, p < 0.01, mouse genotype, F(1, 80) = 0.01, p = 0.94, virus gene × mouse genotype, F(2, 80) = 0.46, p = 0.64, Fig. 6). On the other hand, caMAP2K1 expression had no significant effect on METH-induced CPP in *Drd2-Cre* mice (Fig. 6).

## Discussion

In the present study, we investigated the role of MAPK in accumbal MSNs in reward-associated learning and memory. During the acquisition of Pavlovian conditioning, the number of phosphorylated MAPK1/3-positive cells was increased significantly and exclusively in the NAc core by 7-days of extensive training, suggesting a relationship between Pavlovian conditioning and MAPK signaling in the NAc. Correlation analysis revealed a significant correlation between the number of pMAPK1/3-positive cells and the head entry during CS presentation. D1R-MSNs accounted for 41% of pMAPK1/3-positive cells, whereas 28% were D2R-MSNs after the last training session. The learning curve (CS-head entry rate) in Pavlovian conditioning shifted left-forward only in caMAP2K1-transfected *Drd1a-Cre* mice from that in control mice, whereas the manipulation of MAPK signaling in D2R-MSNs had a negligible effect. Although the head entry rate also increased during ITI period for caMAP2K1-transfected *Drd1a-Cre* mice, it was significantly lower compared to the head entry rate in CS presentation period. These results suggest that MAPK signaling in the D1R-MSNs of the NAc core plays an important role in stimulus-reward learning.

The fact that the number of pMAPK1/3-positive cells was significantly increased by the Pavlovian conditioning training on day 7 compared to day 1 would argue against the stimulus-reward learning account, because one can assume that there may be little new learning taking place by the end of the 7-day training session. It has been shown that repeated training for natural reward increases intracellular and surface expression of glutamatergic receptors in the NAc^30^. Repeated Pavlovian conditioning makes CS an effective predictor of reward delivery and thereby the presentation of CS alone may be sufficient to increase MAPK1/3 phosphorylation in the NAc^24^. We have proposed that phosphorylation of MAPK1/3 after phasic dopamine release increases the excitability of D1R-MSNs and subsequently increases their response to excitatory glutamatergic signals which in turn facilitates further phosphorylation of MAPK1/3^20^, hence the level of pMAPK1/3 after the 7-day training session may become higher than the level after the first session^7^. Initially, phasic dopamine release in the mesolimbic dopamine system is triggered by the receipt of reward, but it shifts to a CS that predicts a reward delivery, after associative learning^31^. We speculate that MAPK1/3 phosphorylation on day 1 may be due to reward obtained because there was no significant difference in the level of pMAPK1/3 between unpaired and paired CS-US groups. Our present study also shows that total MAPK1/3 level was not different between unpaired and paired CS-US groups on either day 1 or day 7, which is consistent with a previous report that shows no changes in total MAPK1 level after Pavlovian conditioning session 1 and session 4^24^.

A previous study reported that the repeated presentation of a reward-associated cue increased the phasic release of DA in the NAc during Pavlovian conditioning^6^. Parkinson *et al*.^32^ have reported that excitotoxic lesions in the NAc core impair the expression of the CS-US association in Pavlovian conditioning. The NAc core is an important substrate for the processing of a reward predictive cue, while the NAc shell is involved in the processing of a non-reward predictive cue^33^. In the present study, the *Drd1a-Cre* mice that had previously expressed higher MAPK activity induced by the AAV-mediated expression of caMAP2K1 in the NAc core showed significantly faster learning of Pavlovian conditioning than control and wtMAP2K1-transfected *Drd1a-Cre* mice. Systemic blockade as well as genetic inactivation of D1R reduced Pavlovian conditioning^1,2^. Activation of D1R potentiates NMDAR activity^34^ and the induction of long-term potentiation (LTP) onto D1R-MSNs depends on D1Rs^35^. Concomitant activation of D1Rs and NMDARs causes DARPP-32 and MAPK phosphorylation and expression of LTP^36–38^. Taken together with these previous findings, our results suggest that activation of MAPK signaling in D1R-MSNs of the NAc core is required for DA-dependent neuronal plasticity associated with stimulus-reward learning.

In the last Pavlovian conditioning session (day 7), all animals approached the reward because they learned the value of reward-predictive CS (goal-tracking). However, some animals attribute incentive salience to CS itself and approach CS upon presentation (sign-tracking)^39^. Thus, CS may simply serve as a predictor of rewards in goal-tracking animals, while CS acquires motivational properties in sign-tracking animals. DA signaling in the NAc is necessary for the acquisition of sign-tracking^6^. Blocked DA signaling by DA receptor antagonists during Pavlovian conditioning training sessions impairs sign-tracking in a drug free test session but not goal-tracking, which indicates necessity of DA signaling for acquisition of sign, but not goal-tracking^6^. In the present study, caMAP2K1-transfected *Drd1a-Cre* mice developed a significant increase in sign-tracking behavior, whereas goal-tracking behavior was similar among all groups. Manipulation of MAPK signaling in D2R-MSNs had negligible effect on sign-tracking and goal-tracking. Thus, MAPK signaling in D1R-MSNs of the NAc facilitates DA-dependent stimulus-reward learning in which incentive salience is assigned to reward cues.

The manipulation of MAPK signaling in D1R- and D2R-MSNs in the NAc had no effect on instrumental learning. It is possible that MAPK signaling in other brain regions such as dorsal striatum may be involved in instrumental task performance. A previous study reported that the level of phosphorylated MAPK1 increased in both the dorsomedial and dorsolateral striatum during acquisition and performance of the instrumental task^40^. Pharmacological blockade of MAPK1 in the striatum impaired acquisition and performance in the instrumental task^24^. Genetic inactivation of D1R in mice results in the loss of motivation to perform instrumental tasks and a deficit in Pavlovian learning, which illustrates the necessity of D1R signaling in these aspects of animal behavior^18^. Both, genetic and pharmacological studies have shown the involvement of D2R in controlling motivation. For example, hyperactivity of D2R leads to a motivational deficit in mice, which may be rescued by decreasing the activity of overexpressed D2R^41^. D2R antagonists have also been shown to facilitate motivation^42^ and increase the phosphorylation of MAPK1/3 in the striatum^43^. In the present study, caMAP2K1-transfected *Drd1a-Cre* and *Drd2-Cre* mice showed increased break points in the progressive task. Thus, striatal MAPK signaling in D1R- or D2R-MSNs may play a crucial role in instrumental learning, while the activation of MAPK signaling in the D1R- and D2R-MSNs of the NAc appear to control the motivational state.

CPP assess the reinforcing effect of drugs using a classical or Pavlovian conditioning principle. In this test, animals learn to associate reward with distinct conditioned cue. Repeated exposure to natural reward and psychostimulants produces synaptic changes in the NAc^30^ but psychostimulants have much stronger reward than natural reward. The rewarding effect of natural reward used in the present study (normal food pallet) may not be so strong as those of psychostimulants or even other natural rewards like sex, flavored food, fatty food, etc. METH-associated CPP analysis provides some insights into a role of MAPK signaling in the NAc under the influence of a much stronger reward than natural food. The results also suggest that MAPK signaling in D1R-MSNs of the NAc facilitates reward associated learning while the signal in D2R-MSNs have little effects.

In conclusion, our results indicate that MAPK signaling in the D1R-MSNs of NAc core contributes to reward-associated learning, while this intracellular signaling in D1R- and D2R-MSNs modulates motivation. Taken together with previous studies, our findings imply that therapies that enhance MAPK signaling in the NAc may be successfully applied to psychiatric disorders that involve learning deficits and motivational dysfunction. Further studies to identify the neural circuits involved in and to elucidate the molecular mechanisms mediating the effects of MAPK signaling on learning and memory, as well as motivation, may reveal novel pharmacological targets for the modulation of these aspects of behavior.

## Materials and Methods

### Animals

All animal experiments were approved by the Animal Experiments Committee of Nagoya University Graduate School of Medicine and performed in accordance with the guidelines for the care and use of laboratory animals established by the Japanese Pharmacological Society and the National Institutes of Health. Detailed information on the generation of mouse strains and sources are reported in Nagai *et al*.^20^. In brief, we used *Drd1a-Cre* (Mutant Mouse Research and Resource Center, UC Davis, CA, USA), *Drd2-Cre* (MMRC), *Drd1a-YFP* (RIKEN BRC, Tsukuba, Japan), *Drd2-YFP* (RIKEN BRC) and C57BL/6 (SLC, Shizuoka, Japan) male mice. Mice were housed under a 12-hour light/dark cycle (light phase 9:00–21:00) at a constant temperature of 23 ± 1 °C and were allowed *ad libitum* food and water access. In food reward-associated behavioral experiments, mice were restricted food to achieve 85% of their *ad libitum* bodyweight. Food restriction was continued throughout the experimental period.

### Drugs

METH hydrochloride was purchased form Dainippon Sumitomo Pharma Co., Ltd. (Osaka, Japan). Haloperidol was purchased from Mitsubishi Tanabe Pharma (Osaka, Japan). In order to exclusively stimulate the endogenous phosphorylation of MAPK1/3 in D1R-MSNs or D2R-MSNs, *Drd1a-Cre* mice received an acute intraperitoneal injection of 10 mg/kg METH 30 min before perfusion, while *Drd2-Cre* mice received 0.5 mg/kg haloperidol 15 min before perfusion. In the CPP test, 2 mg/kg METH was used as a drug reward.

### Stereotaxic injection of AAVs

The AAVs used in the present study and surgeries were described previously by Nagai *et al*.^20^. Mice were anesthetized with tribromoethanol (250 mg/kg, i.p.) and positioned in a stereotaxic apparatus (David Kopf, Tujunga, CA, USA). In order to induce the expression of MAPK signaling in D1R-MSNs, AAV-CAGGS-Flex-EGFP-P2A-wtMAP2K1 or AAV-CAGGS-Flex-EGFP-P2A-caMAP2K1 and to make control group, AAV-CAGGS-Flex-EGFP (1 × 10^12^ genome copies/ml) was injected into the NAc of *Drd1a-Cre* transgenic mice. *Drd2-Cre* mice were transfected with viral vectors containing the mCherry cDNA sequence instead of EGFP. Virus solution (0.5 μl/site) was delivered at four sites (AP: +1.6, ML: ±1.5 and DV: −4.4; and AP: +1.0, ML: ±1.6 and DV: −4.5) at an angle of 10°. All coordinates were relative to the bregma. All experiments were performed 3 weeks after the AAV injection. The data obtained from the animals in which the tip of the glass capillary ended out of the NAc were excluded.

### Immunohistochemistry

Mice were anesthetized with tribromoethanol (250 mg/kg, i.p.) and transcardially perfused with isotonic 4% paraformaldehyde (PFA). The brain was removed, post-fixed in 4% PFA overnight at 4 °C and then cryoprotected in 20–30% sucrose in 0.1 M phosphate buffer. The brain was frozen on dry ice and embedded in Tissue-Tek O.C.T. compound (Sakura Finetechnical). Sections (thickness of 25 μm) between +0.86 to +1.5 (relative to the bregma, the A–P axis) were fixed with 4% PFA and washed with 0.3% Triton X-100/PBS. They were incubated for 1 h in blocking serum (5% normal donkey serum in 0.3% Triton-X 100/PBS) and for 24 h in the presence of mouse monoclonal anti-ERK1 + ERK2 (ab50011) (1:400, Abcam, Cambridge, UK). Sections were then incubated in goat anti-mouse IgG Alexa 568 (1:500, Life Technologies) for 1 h. For total ERK detection rabbit polyclonal anti ERK1 + ERK2 (ab17942) (1:150, Abcam) was used. Regarding YFP and EGFP, sections were incubated for 24 h in the presence of rabbit polyclonal anti-GFP (1:1000, MBL, Nagoya, Japan) followed by a 1 h incubation in goat anti-rabbit IgG Alexa 488, 1:500 (Invitrogen). Regarding mCherry, sections were incubated for 24 h in the presence of rabbit polyclonal anti-RFP (1:500, MBL) followed by a 1 h incubation in goat anti-rabbit IgG Alexa 568, 1:500 (Life Technologies). Samples were observed with a confocal microscope (model LSM780, Carl Zeiss, Jena, Germany). pMAPK1/3 or YFP-positive cells were quantified with Metamorph software (Molecular devices, California, USA) in three sections per animal. In the quantification of pMAPK1/3-positive cells, 300 μm × 600 μm areas were selected in the NAc core and NAc shell. Selected area was isolated and magnified and the number of target cells in each ROI was counted manually by using manually count objects option of the Metamorph software. The results from two ROIs were added to show the total number of positive cells in the NAc core or NAc shell.

### Pavlovian conditioning

The apparatus used for this task consisted of a single compartment (15.9 cm wide × 14.0 cm depth × 12.7 cm high) featuring two stimulus light modules, two illuminated nose poking holes and one pellet receptacle (Med Associates Inc., Georgia, USA). Training was performed as described previously^2^ with minor modifications. We used the nose-poking system instead of lever pressing. Briefly, mice received 7 training sessions and each session contained 25 trials. Each trial was 60s long. In each trial, cue and nose poking hole lights were illuminated for 10 s (CS) followed by the delivery of a 20-mg food pellet (Bio-serv, New Jersey, USA) accompanied by the termination of CS presentation (paired CS-US). In unpaired CS-US control group, timing of food pellet delivery changed randomly within a 60-s trial throughout the session. Thus, control mice received the conditioning training without CS and food reward contingency, but they received equal number of training sessions, food reward and trials per session.

In this test, the CS head entry rate = total number of head entry into the food magazine during CS presentation/(number of CS presentation × duration of each CS); ITI head entry rate = total number of head entry into the food magazine during ITI period/(number of ITI period × duration of each ITI). The final training session was videotaped and the head entry into the food magazine and approach towards the cue light during the CS presentation period was analyzed according to the parameters defined by Gore and Zweifel^2^.

### Instrumental conditioning

Pavlovian conditioning was followed by the fixed ratio 1 (FR1) and FR2 fixed side nose-poking task and then by the FR2 random nose-poking task to receive a single food pellet, which in general is termed as instrumental conditioning. In Pavlovian conditioning, mice do not have to perform any specific task to achieve food reward. However, in instrumental task, mice are required to perform nose poking task achieve a single food pallet. The criteria for each schedule were a 75% correct response rate in active nose-poking holes for three consecutive sessions. FR1 fixed side instrumental conditioning required a single nose poke in the active hole to deliver a single food pellet followed by a 5-s ITI. In the FR2 fixed side task, two nose pokes in the active hole were required to obtain one food pellet. In the FR2 random task, the active nose-poking hole was changed in a pseudorandom manner. Training sessions were terminated until 50 trials or 2 h elapsed. Training in the FR2 random schedule was performed until the correct response rate was ≥75% for three consecutive sessions or for 21 training sessions. Training was extended for one or two extra sessions only if mice showed a correct response rate of ≥75% in the last training session. FR2 random training schedule was analyzed at 3 data points, early, middle and late. Early and late data points are the first and the last training sessions respectively. Middle days are defined for even numbered training schedules as n/2 (n = number of training sessions). For odd numbered training schedule middle days are defined as [(n + 1)/2] (n = number of training sessions). Mice were then subjected to the progressive ratio task. In this task, one reward pellet was delivered upon the completion of each trial in which the nose-poking requirement increased with a non-arithmetic schedule (1, 1, 4, 7, 13, 19…). The breakpoint was the last completed trial before 3 min of nose-poking inactivity or a total 4-h session time-out.

### CPP

The CPP test was performed as described previously by Nagai *et al*.^20^. The apparatus used for this task consisted of two compartments (Brainscience Idea, Osaka, Japan): white and black plexiglass boxes (16 cm wide × 16 cm depth × 17 cm high). In order to enable mice to easily distinguish between the two compartments, the floors of the white and black boxes were covered with transparent rough-surfaced plexiglass and smooth black plexiglass, respectively. A sliding wall isolated each box. In the pre-conditioning stage, the sliding wall had two openings that allowed the mice to move freely between both boxes for 30 min twice a day. During the second session, the time spent in each of the boxes was measured for the first 15 min using MED-PC IV software (MED Associates, Fairfax, Vermont, USA). Pre-conditioning was performed for one day only and was followed by the conditioning period. During the conditioning session, the sliding wall had no opening to restrict mice within one compartment. Conditioning was counterbalanced between compartments to equalize the initial preferences for each compartment within each group. Mice were conditioned with saline (i.p.) in one chamber in the morning sessions for 30 min and 2 mg/kg METH (i.p.) in the other chamber in the afternoon sessions for 3 days. Control mice received saline in both sessions. On day 5, the post-conditioning test session was performed. Mice were allowed to move freely between the boxes for 15 min for a single session and time spent in each box was recorded. The CPP score for each mouse was calculated by subtracting the time spent in the pre-conditioning stage from that of the post-conditioning stage. Mice that showed a preference for one side in the pre-conditioning session was excluded for the final CPP score calculation.

### Statistical analysis

All data are expressed as the mean ± SEM. A one-way or two-way analysis of variance (ANOVA) was used followed by Tukey’s test when the F ratios were significant (p < 0.05). The significant of differences between two groups was analyzed using the Student’s *t*-test. Pearson correlation coefficient analysis was used for correlation between pMAPK1/3 and behavioral performance.

## Electronic supplementary material

Supplementary information

## Data Availability

Data generated or analyzed during this study are included in whole or in part in this published article. Where only part of a data set is included, reasonable requests can be made to the corresponding author.
