# Supplementary material for: Cell type-specific activation of mitogen-activated protein kinase in D1 receptor-expressing neurons of the nucleus accumbens potentiates stimulus-reward learning in mice
Source: Sci Rep. 2018 Sep 26;8:14413. doi: 10.1038/s41598-018-32840-1 (PMC6158283; doi:10.1038/s41598-018-32840-1)
Supplement: Supplementary file 1 — Supplementary information [file 41598_2018_32840_MOESM1_ESM.pdf]

Cell type-specific activation of mitogen-activated protein kinase in D1 receptor-expressing neurons of the nucleus accumbens potentiates stimulus-reward learning in mice

Authors: Md. Ali Bin Saifullah[1,#], Taku Nagai[1,#,*], Keisuke Kuroda[2], Bolati Wulaer[1], Toshitaka Nabeshima[3], Kozo Kaibuchi[2], Kiyofumi Yamada[1,*]

Affiliations: [1]Department of Neuropsychopharmacology and Hospital Pharmacy, Nagoya University Graduate School of Medicine, Japan, 466-8560. [2]Department of Cell Pharmacology, Nagoya University Graduate School of Medicine, Japan, 466-8560. [3]Advanced Diagnostic System Research Laboratory Fujita Health University, Graduate School of Health Sciences, Toyoake, Japan, 470-1192.

#Contributed equally to this work

* Address for correspondence:

Kiyofumi Yamada, Ph.D. and Taku Nagai, Ph.D., Department of Neuropsychopharmacology and Hospital Pharmacy, Nagoya University Graduate School of Medicine, 65 Tsurumai-cho, Showa-ku, Nagoya 466-8560, Japan.

Tel.: +81-52-744-2674; Fax: +81-52-744-2979

E-mail address: kyamada@med.nagoya-u.ac.jp or t-nagai@med.nagoya-u.ac.jp

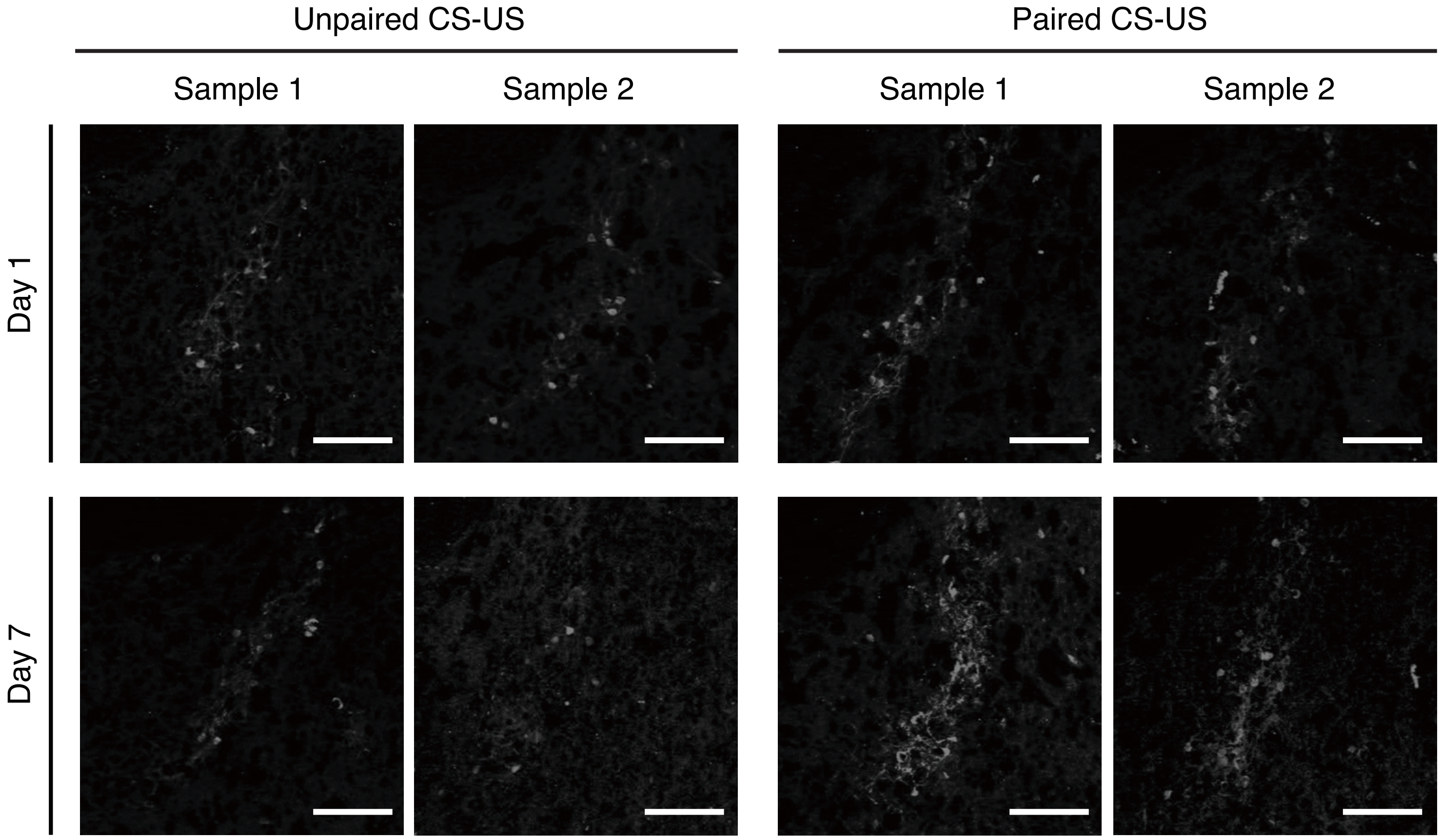

Figure S1. Phosphorylation of MAPK1/3 in the NAc during acquisition of the Pavlovian conditional approach
Confocal images of pMAPK1/3-positive cells in the NAc core after training session in the unpaired CS-US and paired CS-US group after training session on day 1 and day 7. Scale bars represent 100 µm.

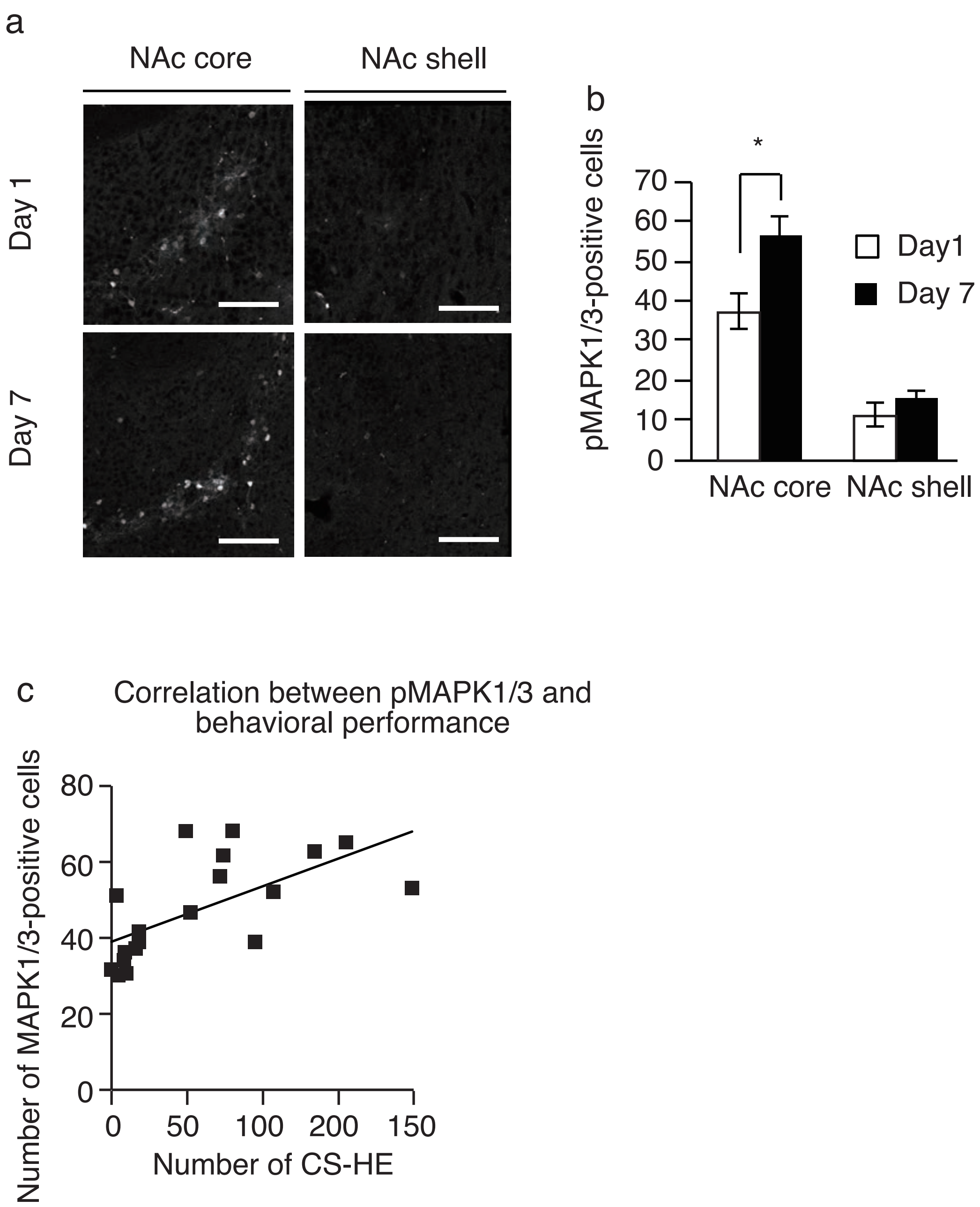

Figure S2. Number of pMAPK1/3 and correlation between pMAPK1/3 in the NAc shell
(a) Confocal images of pMAPK1/3-positive cells in the NAc after the conditioning session. Scale bars represent 100 µm. (b) Quantification of pMAPK1/3-positive cells in the NAc core and shell (n=4 for day 1, n=6 for day 7). Data are presented as the mean ± SEM. *p<0.05. (c) Correlation between pMAPK1/3 count and behavioral performance during Pavlovian conditioning in C57BL/6 mice (n=8 for Day 1, n=11 for Day 7).

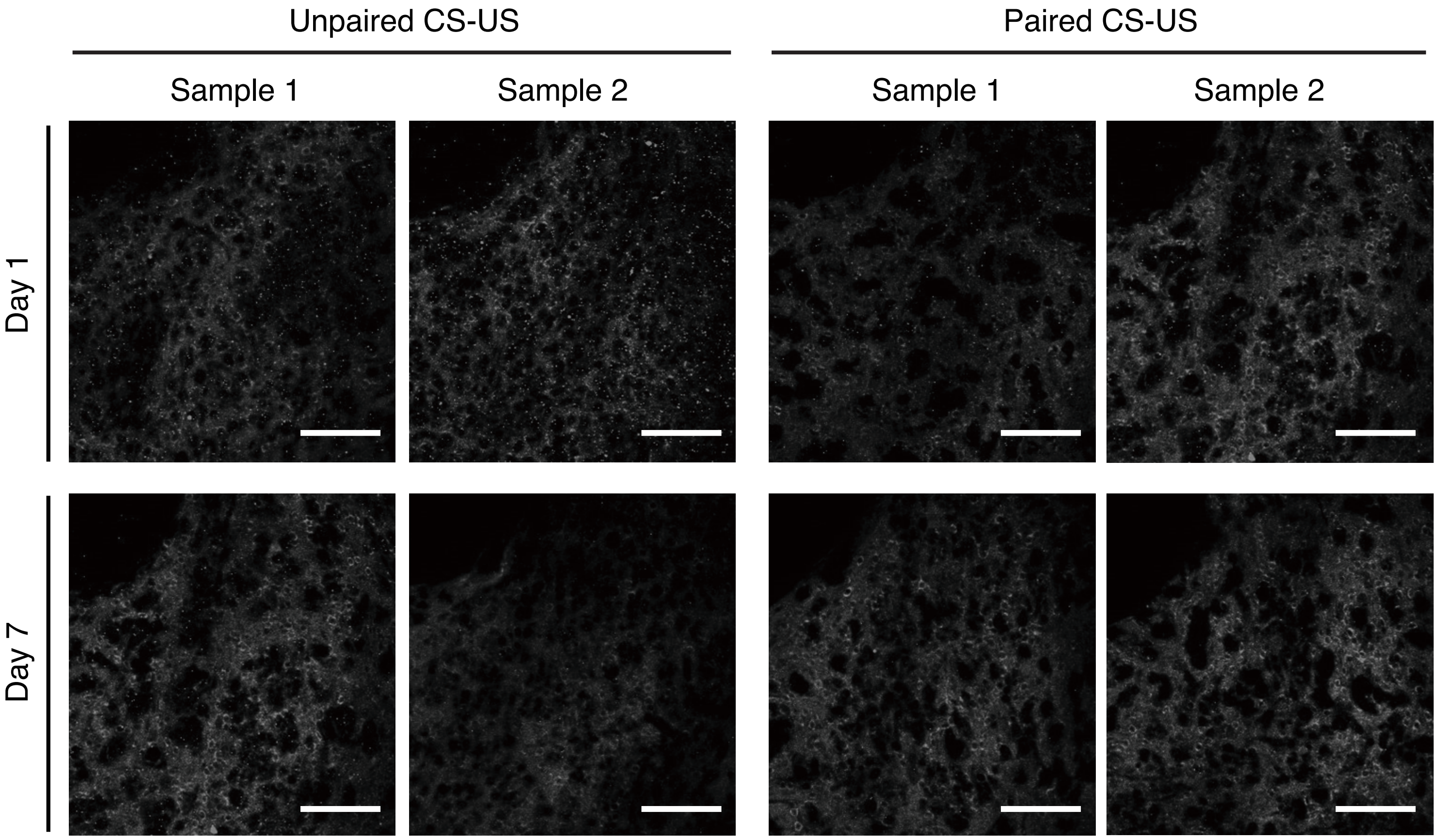

Figure S3. Total MAPK1/3 in the NAc during acquisition of the Pavlovian conditional approach

Confocal images of total MAPK1/3-positive cells in the NAc core after training session in the unpaired CS-US and paired CS-US group after training session on day 1 and day 7. Scale bars represent 100 µm.

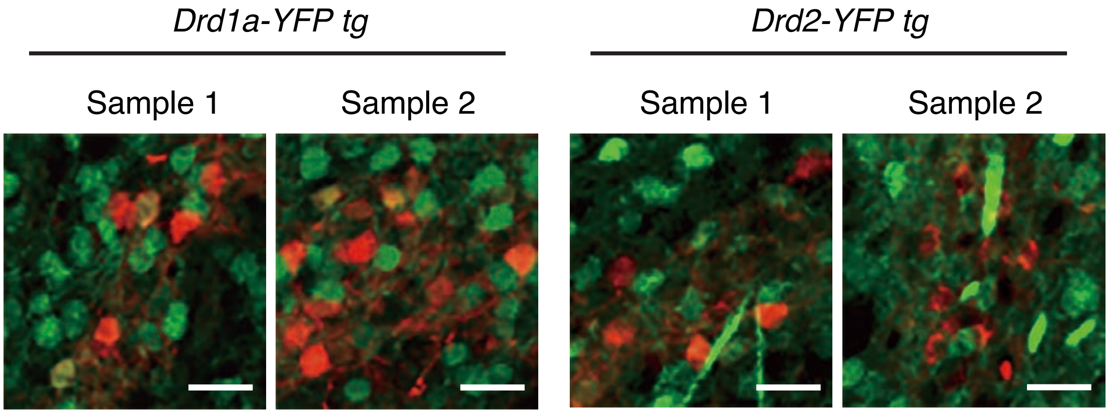

Figure S4. Proportion of phosphorylated MAPK1/3-positive cells in the NAc core of Pavlovian-conditioned *Drd1a-YFP* and *Drd2-YFP tg* mice

Representative confocal images of pMAPK1/3-positive cells in the NAc core of *Drd1a-YFP and Drd2-YFP tg* mice after the training session of Pavlovian conditioning on day 7. YFP-positive (green) and pMAPK1/3-positive (red). Scale bars represent 25 μm.

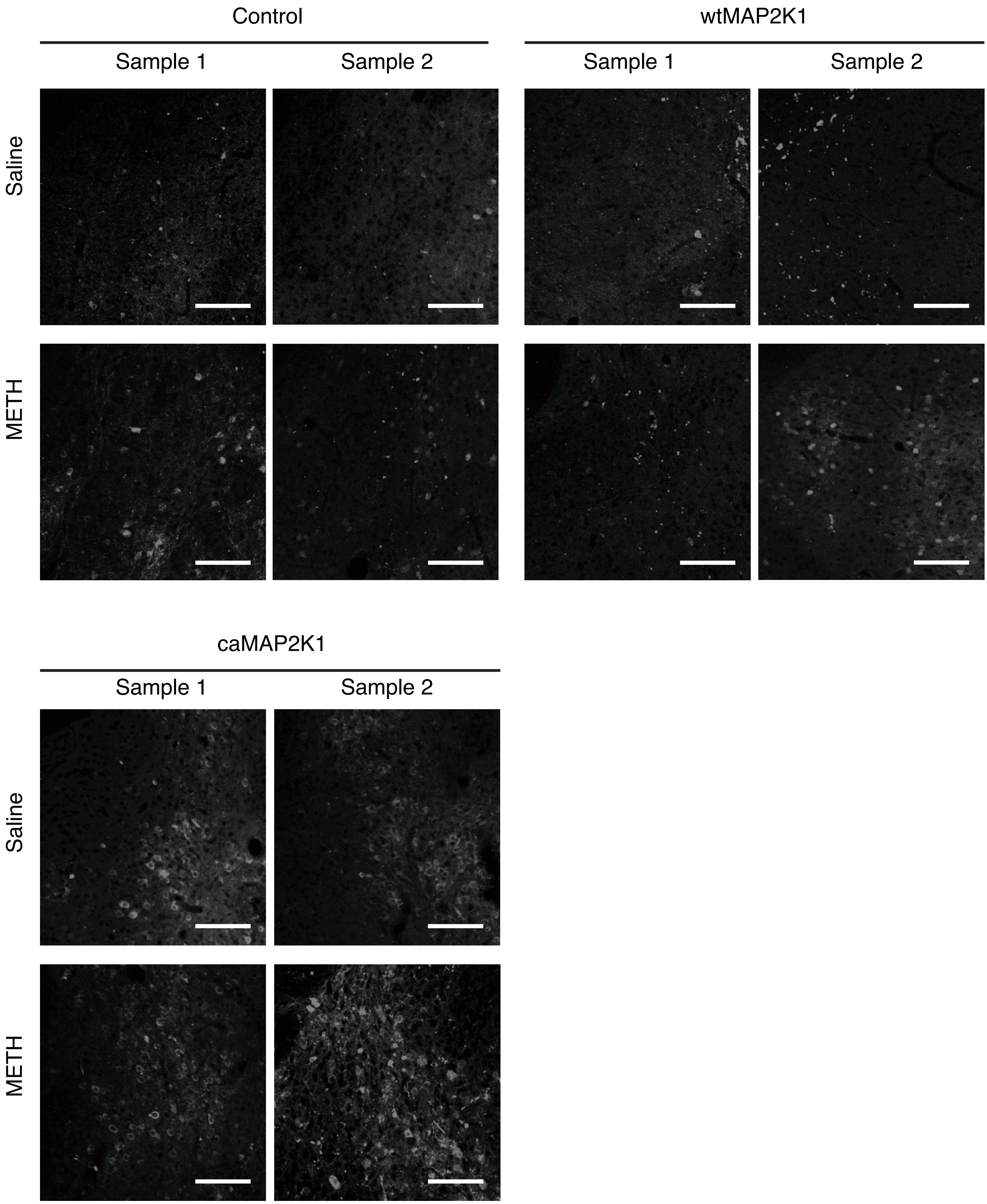

Figure S5. Manipulation of MAPK signaling in accumbal D1R-MSNs
Representative confocal images of pMAPK1/3-positive cells in the NAc of AAV-mutant MAP2K1-injected *Drd1a-Cre tg* mice. *Drd1a-Cre tg* mice were microinjected with AAV-mutant MAP2K1 into the NAc. Three weeks after the treatment, mice were administered saline or METH (10 mg/kg, i.p.) 30 minutes before perfusion. The upper panel represents saline-treated mice and the lower panel represents METH-treated samples in each group of mice. Scale bars represent 100 µm.

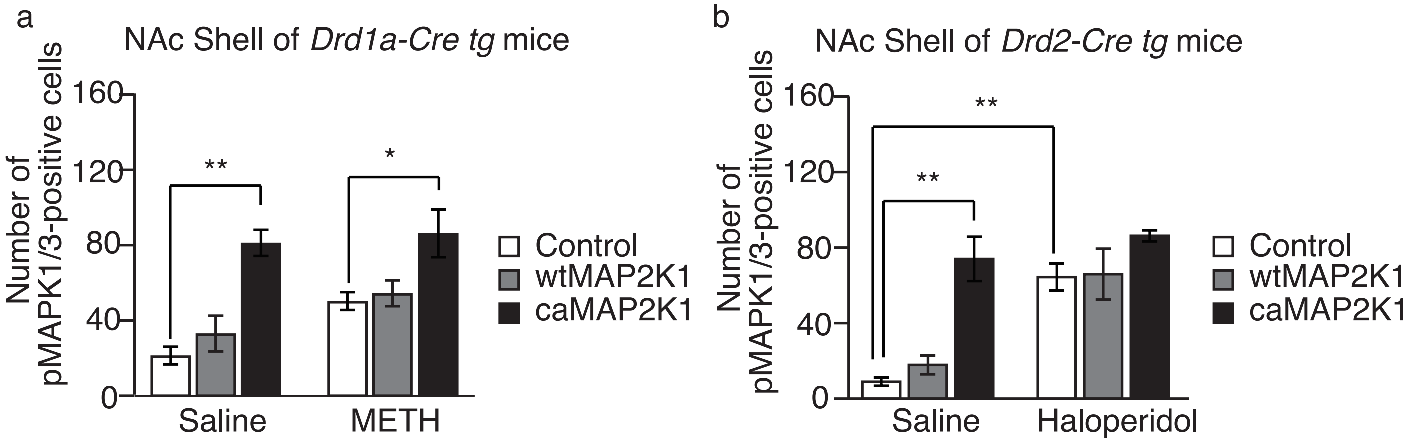

Figure S6. pMAPK1/3-positive cells in the NAc shell of *Drd1a-Cre* and *Drd2-Cre tg* mice
(a) Quantification of pMAPK1/3-positive cells in the NAc shell of *Drd1a-Cre tg* mice (n=5 for saline-treated control, n=3 for saline-treated wtMAP2K1, n=3 for saline-treated caMAP2K1, n=4 for METH-treated control, n=3 for METH-treated wtMAP2K1, n=4 for METH-treated caMAP2K1). $^*p<0.05$, $^{**}p<0.01$. (b) Quantification of pMAPK1/3-positive cells in the NAc shell of *Drd2-Cre tg* mice (n=5 for the saline-treated control, n=3 for saline-treated wtMAP2K1, n=3 for saline-treated caMAP2K1, n=4 for the haloperidol-treated control, n=3 for haloperidol-treated wtMAP2K1, n=3 for haloperidol-treated caMAP2K1). Data are presented as the mean ± SEM. $^{**}p<0.01$.

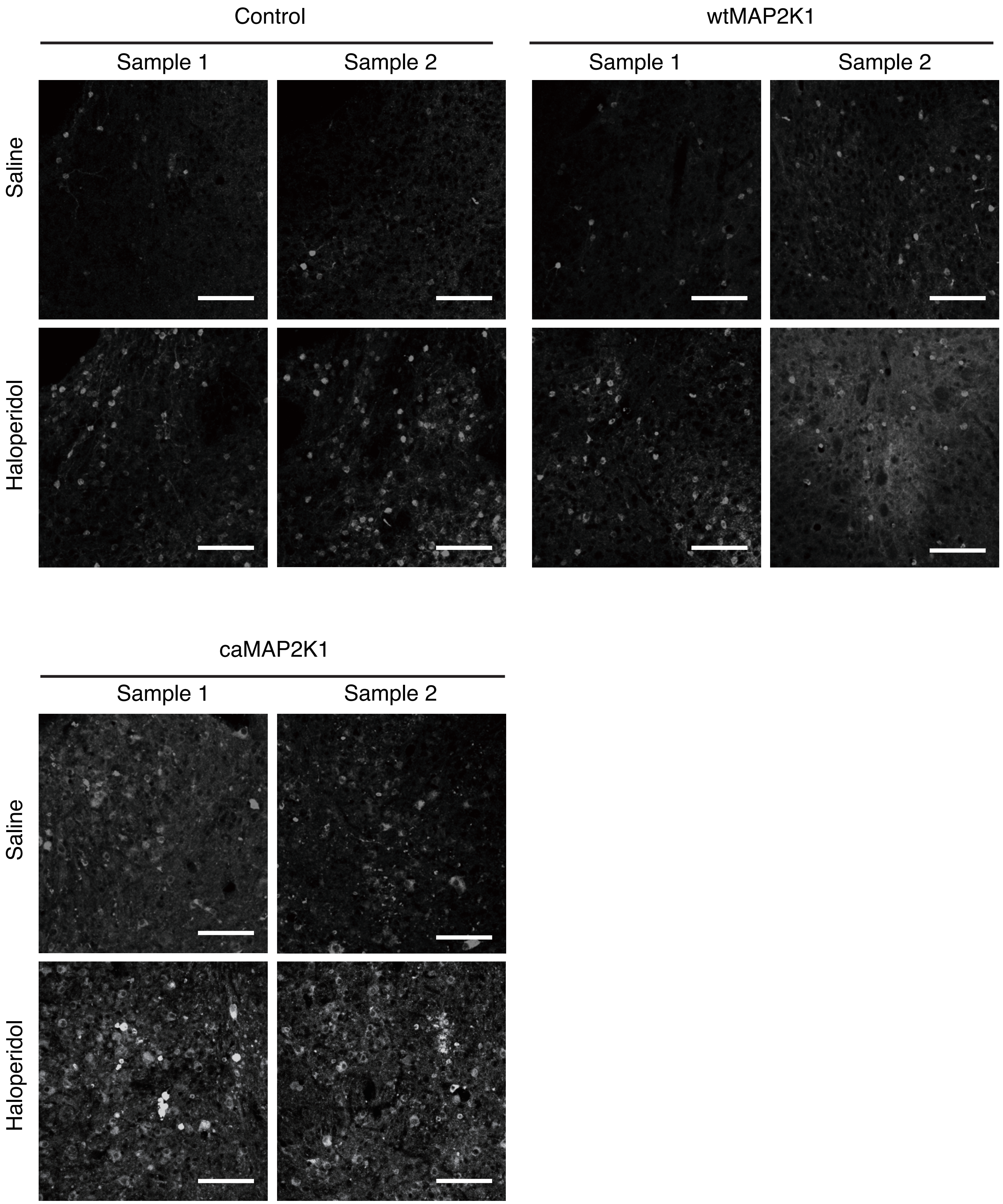

Figure S7. Manipulation of MAPK signaling in accumbal D2R-MSNs
Representative confocal images of pMAPK1/3-positive cells in the NAc of AAV-mutant MAP2K1-injected *Drd2-Cre tg* mice. *Drd2-Cre tg* mice were microinjected with AAV-mutant MAP2K1 into the NAc. Three weeks after the treatment, mice were administered saline or haloperidol (0.5 mg/kg, i.p.) 15 minutes before perfusion. The upper panel represents saline-treated mice and the lower panel represents haloperidol-treated mice in each group. Scale bars represent 100 µm.

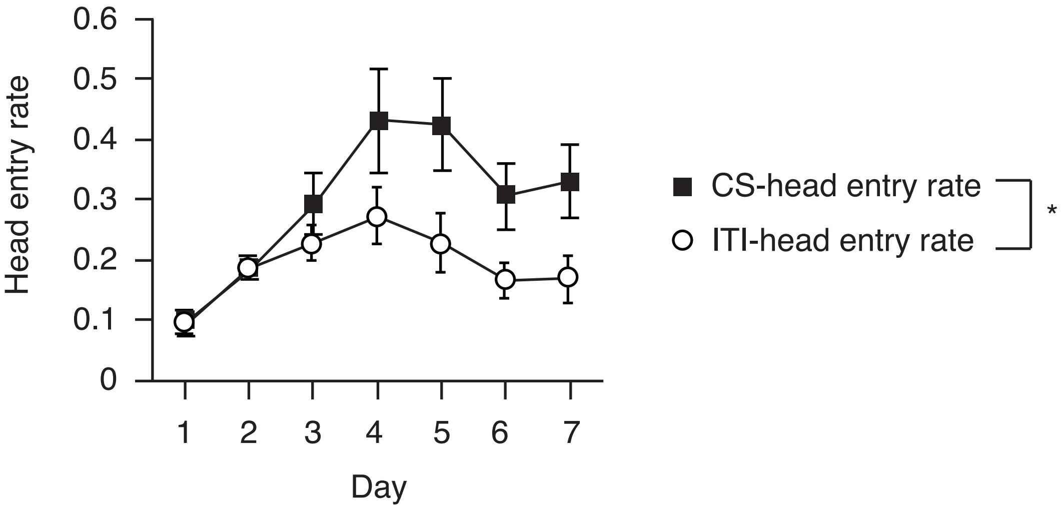

Figure S8. Performance of caMAP2K1-transfected Drd1a-Cre tg mice during acquisition of the Pavlovian conditional approach

Head entry rate during CS and ITI in caMAP2K1-transfected *Drd1a-Cre tg* mice (n=8). Data are presented as the mean ± SEM. *p<0.05.

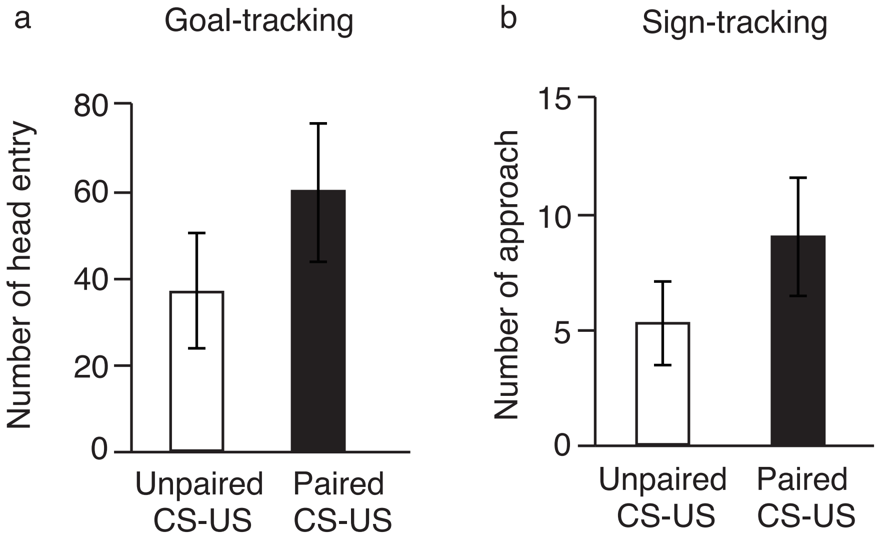

Figure S9. Goal-tracking and sign-tracking behaviors in C57BL/6 mice
(a) Goal-tracking and (b) sign-tracking in C57BL/6 mice (n=7 for unpaired CS-US, n=6 for paired CS-US group).
